# Improving the Nutritional Status of Socially Vulnerable Children in Manaus, Brazilian Amazon, through a Food Supplementation Programme

**DOI:** 10.3390/nu16071051

**Published:** 2024-04-04

**Authors:** Elaine Martins Bento Mosquera, Karina Merini Tonon, Rosângela Aparecida Augusto, Tânia Maria de Carvalho, Mauro Batista de Morais

**Affiliations:** 1 Nutrition Postgraduate Program, Universidade Federal de São Paulo, São Paulo 04530-050, SP, Brazil; elainembento@gmail.com; 2 Medical Engagement Nestlé, São Paulo 07730-090, SP, Brazil; 3 Independent Researcher, Florianópolis 88034-100, SC, Brazil; 4 Independent Researcher, São Paulo 02405-100, SP, Brazil; augusto.ro@gmail.com; 5 Nutritionist at the Manaus’ State Health Office, Manaus 69049-110, AM, Brazil; tbatista7@hotmail.com; 6 Division of Pediatric Gastroenterology, Universidade Federal de São Paulo, São Paulo 04020-040, SP, Brazil

**Keywords:** child nutrition sciences, nutritional status, dietary supplementations, programme evaluation, cohort studies

## Abstract

Information on the effects of government nutrition programmes provided to socially vulnerable children to improve their nutritional status is scarce. We analysed the effectiveness of a nutritional programme, including food supplementation with infant formula, on the evolution of the weight and height of socially vulnerable children from Manaus in the Brazilian Amazon. This study included 7752 children aged 12–24 months admitted to the programme between 2017 and 2020. Weight and height measurements at admission and every three months thereafter were extracted from the programme database. Weight-for-age, weight-for-height, body mass index-for-age (BMI/A), and height-for-age z-scores were analysed using a multilevel linear regression model, which showed a statistically significant decrease in nutritional deficits toward nutritional recovery at follow-up. The programme’s effectiveness was evaluated in 1617 children using a paired analysis comparing data from between 12 and 15 months of age at admission and follow-up after 6–9 months. Children admitted with wasting presented an increase in the BMI/A z-score, whereas children admitted with a risk of being overweight and obese had a statistically significant decrease in the BMI/A z-score. Children admitted with stunted growth also showed increased height-for-age z-scores. The nutrition programme was effective for children experiencing wasting and reducing excess weight.

## 1. Introduction

Worldwide data from the United Nations International Children’s Emergency Fund [1] show that more than 149 million children under five years old have chronic malnutrition, defined by a height-for-age deficit. By contrast, 45 million patients have acute malnutrition (i.e., a weight deficit), and nearly 39 million are overweight [1]. The eating patterns of infants and children affect their growth and development, especially in the first years of life [2]. The World Health Organization (WHO) recommends exclusive breastfeeding until the sixth month of life and complemented breastfeeding at least for two years [3,4,5]. However, global and Brazilian surveys have shown that only 45% of infants receive exclusive breastfeeding until five months of age [6]. For infants who cannot be breastfed or receive donor human milk, adequately prepared infant formula is recommended to meet the nutritional requirements of their age [7]. Early-weaned children from families living in socially vulnerable areas are subject to inadequate complementary food and have a higher risk of wasting and stunting [8]. 

Manaus is the most populous city in the Amazonas and the seventh-largest city in Brazil, with the largest number of inhabitants [9]. In terms of health, the child population is one of the most vulnerable to nutritional deficits; however, it also has nutritional diversity. A previous study evaluating nutritional status found that children who attended public daycare centres were underweight for both age and height, while those who attended private daycare centres tended to be overweight [10,11,12,13].

Governments worldwide employ food supplementation programmes to prevent nutritional deficiencies in vulnerable populations. However, these programmes are often not monitored or evaluated. Systematic reviews have investigated various nutrition-based approaches designed to improve child growth and development, including the promotion of breastfeeding, iron supplementation, multiple micronutrient supplementation, complementary feeding education, integrated community-based nutrition and stimulation interventions, supplementary feeding, and therapeutic foods for acute malnutrition [14,15,16]. However, few studies have evaluated the impact of nutrition programmes, including milk or infant formula, on the evolution of weight and height of socially vulnerable children [17,18,19,20,21,22,23,24].

Therefore, this study aimed to evaluate a nutrition programme that included infant formula supplementation to improve the nutritional status of socially vulnerable children aged 12–24 months living in Manaus, Brazil.

## 2. Materials and Methods

### 2.1. Study Design

This real-world retrospective cohort study evaluated secondary data from children living in Manaus, Brazil, who joined the city’s food supplementation programme ‘Leite do Meu Filho’, which is aimed at low-income families. Data were obtained from the database adopted by the Manaus Municipal Health Office for the management and monitoring of participants.

### 2.2. Participants

This study evaluated the evolution of weight and height in 7752 children aged 12–24 months. Data were obtained from a database containing 20,247 children aged 12–48 months admitted to the nutrition programme between January 2017 and February 2020. The criteria for entering the study were having complete anthropometric data and more than one measure in addition to that obtained at admission. The exclusion criteria are shown in Figure S1.

### 2.3. Food Supplementation Programme ‘Leite do Meu Filho’

The programme ‘Leite do Meu Filho’ was created in 2011 by the Municipal Health Office of Manaus to secure the adequate nutritional status of children up to 4 years and 11 months old from families living in social vulnerability. Children registered in the programme receive quarterly guidance and nutritional status and development monitoring by primary care professionals. This includes family physicians, paediatricians, nurses, nutritionists, and social workers at the basic health unit closest to the family’s residence. 

The programme also provides food supplements. Children aged 12–24 months receive 1.6 kg/month of powdered infant formula. Polymeric and normo-caloric infant formula contains intact proteins, lactose, prebiotics, iron, vitamin D, and other minerals and vitamins. The complete compositions are presented in Table S1.

During the first evaluation of the children upon admission to the programme, information such as sex, date of birth, gestational age, birth weight, hospitalisation, vitamin A and iron supplementation, weight, and height was obtained. Additionally, information was collected on the mother’s age (years) and the number of children. At each follow-up visit, clinical data, anthropometric measurements, and consumption markers were obtained through a food frequency questionnaire and an assessment of the development of the children participating in the programme. Trained health professionals performed the anthropometric measurements using a calibrated scale, anthropometric tape, and a stadiometer. All the clinical and nutritional data were entered into a database managed by the Municipal Health Office of Manaus.

### 2.4. Assessment of the Nutritional Status

The following z-scores were calculated: weight-for-age (W/A), weight-for-height (W/H), body mass index-for-age (BMI/A), and height-for-age (H/A). The WHO Anthro (2007) software based on WHO growth curves was used for the z-score calculation [25]. The ‘FLAG’ code was used to identify children with measurements not included in the range recommended for z-scores, that is, between −5 and +5 standard deviations (SD). The weight and height measurements obtained at admission were used as a baseline.

### 2.5. Data Analysis

The effectiveness of the ‘Leite do Meu Filho’ programme in improving children’s nutritional status was evaluated using two statistical strategies: (1) multiple linear regression model (multilevel) and (2) paired evaluation between admission (before the intervention) and after 6–9 months of participation in the programme (during the intervention).

#### 2.5.1. Multiple Linear Regression Model (Multilevel)

Initially, the data of the children admitted with nutritional deficits and the raw data of all indicators (W/A, W/H, BMI/A, and H/A) during their stay in the programme were compared with the admission data using linear regression for continuous data. After a preliminary analysis, a multilevel model was developed. Thus, the weight and height data obtained on admission were considered the main independent variables (0 = admission; 1 = participants in the statistical analysis).

Children’s age at follow-up was used as a continuous variable. After the preliminary analysis, the square of age was included in the multiple analysis for the curves of indicators W/A, W/H, and BMI/A to allow for a better format approximation of the z-score evolution curves with age advancement. Age squared did not show statistical significance for the H/A indicator and was therefore excluded from the model. 

The multilevel model considered the role of supplementation programmes in children with nutritional deficits. Based on the admission evaluation, the sample was divided into two categories: with or without a nutritional deficit (score z < −2). Multilevel modelling provided an assessment of the follow-up of weight and height during the intervention of the data of 7752 children, which included 23,344 measures of weight and height. 

In the multilevel modelling, a variable was generated from the combination of variable status in the programme and nutritional status on admission, resulting in four categories for the evaluation of effectiveness:

- Programme = 0 + nutritional deficit on admission (NDA) = 0: equivalent to the anthropometric evaluation performed on admission to the programme for children without nutritional deficits.
- Programme = 1 + NDA = 0: equivalent to the anthropometric evaluation performed after two months or more of programme participation in children admitted without nutritional deficits.
- Programme = 0 + NDA = 1: equivalent to the anthropometric evaluation performed on admission for children with nutritional deficits.
- Programme = 1 + NDA = 1: equivalent to the anthropometric evaluation performed after two months or more of programme participation in children admitted with nutritional deficits.

Data are presented as proportions, means, and SDs. In the multilevel modelling, regression coefficient estimates (Coef), 95% CI, and descriptive statistics are presented. Processing was conducted using the Stata software (Stata Corp LP 2014, College Station, TX, USA). Modelling was conducted using multilevel multiple linear regression to explore the structure of the hierarchical data analysis. A statistical significance level of α = 5% was adopted. In multilevel modelling, variables related to maternal and child characteristics were not included as the same child was seen more than once over time, and this information was mainly fixed during the observation period, such as weight at birth, sex, delivery type, and gestational age.

The crude effect of the programme was measured for the total sample and separately for each Z-score category upon admission. In the total sample and in each group, all anthropometric evaluations obtained at admission were used as control measures because the child was not yet consuming the offered food supplementation, and these served as a baseline to evaluate the programme’s effectiveness on the children’s nutritional status. 

The effects of the measurements in each nutritional category were compared, considering the group ‘without nutritional deficit on admission’ as the baseline. After exploring the crude model, multilevel modelling adjusted for age at follow-up and stratified for each nutritional condition is also presented. After this stage, multiple multilevel modelling was conducted. With the complete model, adjusted z-score values were obtained for all combinations (age, z-score category at admission, and programme status), allowing the construction and comparison of nutrition-following curves for each indicator.

#### 2.5.2. Paired Evaluation from the Baseline to 6–9 Months after the Admission

This analysis included data from 1617 children who were admitted between 12 and 15 months of age and underwent follow-up weight and height measurements 6–9 months after admission to the nutrition programme. At admission, data were evaluated according to BMI classification: normal, wasting, overweight, overweight, and obese. Stunting (W/H < 2, 0 SD) was assessed. 

A paired *t*-test was used to compare the z-scores at admission and after six to nine months of follow-up. The significance level of α = 5% was adopted.

### 2.6. Ethical Consideration

This retrospective cohort study was approved by the Research Ethics Committee of the Federal University of São Paulo (protocol no. 3.376.656). The study was conducted using data obtained from the ‘Leite do Meu Filho’ database, formally granted by the Municipal Health Office of Manaus (authorisation no. 032/2019-ESAP/SEMSA).

## 3. Results

Table 1 shows the general characteristics of the study population. The mothers’ mean ± SD age was 28.4 ± 6.6 years. Sociodemographic and nutritional data of the children admitted to the programme between 12 and 24 months of age were similar for both sexes. The prevalence of premature birth was 5.5%. The prevalence rates for W/A, W/H, BMI/A, H/A were 1.6%, 1.3%, 1.7%, and 12.6%, respectively.

A total of 23,344 weight and height measurements were conducted, encompassing admission and follow-up measurements, among the 7752 children admitted between the ages of 12 and 24 months. These measurements were conducted with a minimum interval of two months between assessments (Table 2). The mean of the measurements was three per child. 

**Figure 1:**
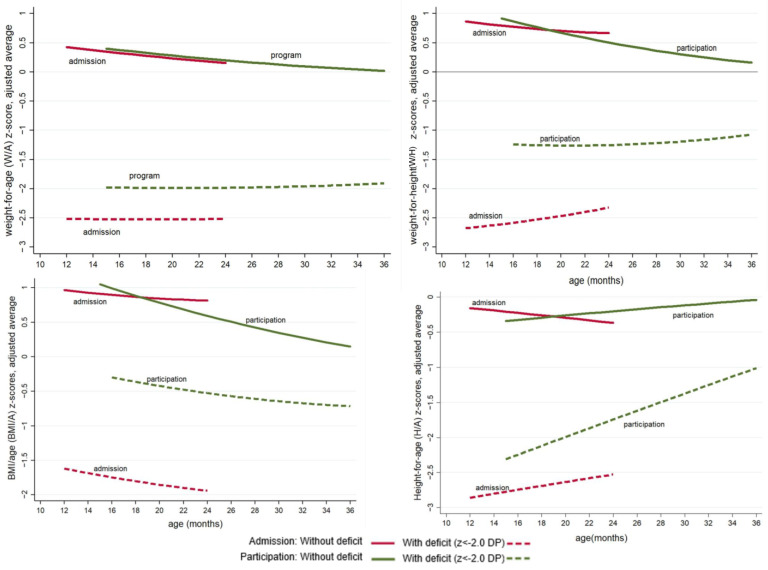
Adjusted average curves of z-scores (W/A, W/H, BMI/A, H/A) according to the nutritional deficit and exposure to the programme (admission and participation; *n* = 7752), Manaus, 2017–2020.

The data shown in Table 3, Table 4 and Table 5 and Figure 1 refer to the multilevel modelling. 

Table 3 presents a crude data comparison of the number of children admitted with a nutritional deficit according to the four indicators and the number of children whose nutritional deficit persisted throughout the intervention, as observed at follow-up. Regarding admission, there was a statistically significant reduction in the prevalence of W/A, W/H, BMI/A, and H/A deficits in children, except after the fourth follow-up for the W/H and BMI/A indicators when only one child remained with z-score deficits.

Table 4 shows a statistically significant reduction in the mean z-scores on admission, except for the W/H z-scores at the fourth follow-up visit and the BMI/A z-scores at the third and fourth follow-up visits. 

Table 5 shows that, in the crude model for the total sample, the mean z-scores for W/A, W/H, and BMI/A at follow-up were significantly lower than the mean obtained on admission, in particular with coefficients equal to −0.0635, −0.1831, and −0.2125 z-score units, respectively. A similar result was also observed in children admitted without deficit with coefficients equal to −0.0739, −0.2025, and −0.2442, respectively. Conversely, children admitted with deficits in W/A, W/H, and BMI/A had positive coefficients of +0.5738, +1.2889, and +1.4073, respectively, suggesting nutritional recovery. Regarding the H/A indicator, a more expressive effect was observed in children admitted with H/A deficits (+0.9371 z-score units). 

The complete model explored programme effects according to nutritional status (presence or absence of a deficit) at admission, as defined by each of the four indicators (W/A, W/H, BMI/A, and H/A). During follow-up, there was a reduction in the z-scores of W/A (−0.0246), W/H (−0.0269), and BMI/A (−0.0205) and an increase in H/A (+0.0177) in children admitted without nutritional deficits. In the group of children admitted to the programme with deficits, the W/A z-scores (+0.0570) and H/E (+0.0452) increased with age and did not change for W/H (+0.0464) or BMI/A (−0.0141). In the participant group with a W/A deficit upon entry, the increase was +0.0253 z-score units (+0.0570–0.0317) for each month of increase in age in the programme. Furthermore, the effect of the programme was more significant at younger ages. 

In the complete model, the W/H and BMI/A z-scores of children admitted without deficits decreased with age during participation in the programme. Therefore, at each month of follow-up, there was a reduction of −0.0315 and −0.0397 (programme × age). In children with deficits, z-scores did not change with increasing age during participation in the programme, as verified by the lack of statistical significance for the programme × age × deficit variable.

In the crude model for H/A, the mean z-scores were significantly higher in the total sample (+0.1496) and in children admitted with a deficit (+0.9371). However, there was no statistically significant difference in the number of children without deficits (+0.0428) upon admission. However, the H/A z-scores varied positively, with a statistically significant reduction in the severity of the admission deficit.

The effect adjusted by the programme on children admitted without z-score deficit is provided by the category coefficient ‘in the programme’ of the variable ‘status in the programme’, being significantly higher for W/A indicators (+0.0694), W/H (+0.2211), and BMI/A (+0.2558) and lower for H/A (−0.2244). The sum of this effect with the variable coefficient programme × deficit resulted in a programme-adjusted effect in the group with the deficit, indicating that the programme had a significantly greater effect on the z-score gain for all studied parameters.

Figure 1 shows the evolution of the z-scores from admission to 12 months of follow-up according to the nutritional deficit at admission. There was a positive impact on W/H and BMI/A z-scores in children admitted with nutritional deficits. The BMI/A tended to decrease as the length of follow-up increased in both groups. However, in the group with the BMI/A deficit at admission, there was an expressive and positive deviation, indicating nutritional recovery. The H/A ratio in both groups showed an upward trend as the follow-up period increased.

The paired evolutions of weight and height after 6–9 months of follow-up are shown in Table 6 and Table 7, according to nutritional status at admission.

In children admitted without anthropometric deficit, there was an oscillation (*p* < 0.05) in all parameters, with mean differences between −0.11 and −0.14 SD (Table 3). In children admitted with anthropometric deficits, there was a statistically significant reduction in deficits with greater nutritional relevance, considering the differences between the mean, which were between +0.68 and +1.61 SD (Table 6).

The mean evolution of the anthropometric scores according to the nutritional status (BMI/A and H/A z-scores) at admission is presented in Table 4. The BMI/A increased in children with wasting and in those with normal BMI/A at admission; however, this increase was more significant in children with wasting. For children admitted with a BMI/A > 1.0 SD, a significant (*p* < 0.05) decrease was inversely proportional to excess weight. 

In the evolution of the nutritional status classified according to BMI/A values, a reduction in the BMI deficit with age was observed in the 25 children admitted with wasting with a difference between the z-score averages of +1.61 SD. The oscillation in the group admitted with adequate weight was also positive, with an average difference of +0.22 SD. Conversely, children admitted at risk of overweight, overweight, and obesity showed reductions in BMI z-scores for age with differences between averages of −0.27, −0.78, and −1.23 SD (Table 7).

## 4. Discussion

This study showed that the programme ‘Leite do Meu Filho’ had a positive impact not only on the reduction of weight and height deficits but also on the reduction of weight excess (overweight and obesity).

Food supplementation programmes with dairy products have been in use for decades, focusing on their contribution to combating child malnutrition and reducing macro- and micronutrient deficiencies. However, few studies have evaluated its effectiveness in positively changing the nutritional status of overweight children [18,26,27,28,29,30,31,32,33,34].

A systematic review and meta-analysis were conducted to evaluate the effects of fortified milk or infant formula versus a control group on the growth and nutritional status of children under five years of age. These results suggest that the growth outcomes are superior in developing economies. In particular, there is a significant impairment due to dietary inadequacy, which promotes a reduction in the risk of anaemia [15]. Another systematic review highlighted that milk fortified with multiple micronutrients in children under three years of age is an effective option for reducing anaemia in developing countries [16]. A recent systematic review and meta-analysis showed that integrated nutritional interventions in places with high malnutrition significantly promoted growth and development [14]. 

A study conducted in Manaus used four anthropometric indicators to obtain greater accuracy in the assessment of nutritional status. Children admitted with nutritional deficits showed a statistically significant increase in the z-scores for the W/A, W/H, H/A, and BMI/A indicators. In contrast, children with excess weight who were admitted to the programme showed a significant reduction in BMI z-scores with age. Therefore, the programme, which provided multidisciplinary assistance and infant formula distribution, corrected the inadequacies in the two extremes of nutritional status: malnutrition and overweight. 

Previous studies have shown that children in the Amazon region have lower anthropometric parameters than the Brazilian mean [35] and that the food consumption of Brazilian children presents nutritional deficiencies, especially in micronutrients [13]. These deficiencies in the first few years of life can result in developmental deficits with metabolic effects, considering the need for specific nutrients and micronutrients critical for growth, such as iron, zinc, copper, iodine, and B vitamins [2,13]. Thus, adopting a nutritional strategy that includes dairy-fortified foods that contribute to the adequate intake of macro- and micronutrients can be crucial in minimising deficiency risks and impacts on child growth and development [36]. 

The evaluation of food supplementation programmes in Brazil differs in terms of methodology, type of food used for supplementation (cow milk, cow milk supplemented with vegetable oil, or infant formula), intervention duration, and anthropometric indicators. Despite the lack of homogeneity, studies have shown that nutrition programmes improve anthropometric indicators, with children with more significant deficits benefiting the most [19,23,24,27,28,33]. Two exceptions were studies that did not show a response to the nutritional intervention programme; there were failures in maternal adherence and home delivery of milk in one study [31]. In another study, ineffectiveness was attributed to significant age variability during study admission [32]. 

A study conducted in the State of São Paulo, Brazil, used the same multilevel regression method to evaluate only the W/A indicator of children admitted to a programme with nutritional deficits [23]. These results converged with those of the malnourished children, positively affecting the anthropometric indicators of beneficial evolution [23]. Thus, the results of the present study agree with most previous studies conducted in Brazil that demonstrated the effectiveness of nutritional interventions. 

Nutrition programmes can contribute positively to the recovery of nutritional status and prevent the occurrence of nutritional deficiencies within the scope of social food, nutrition, and food security policies. In particular, it should be combined with health actions that involve periodic monitoring by primary care professionals, especially in socioeconomically vulnerable populations, as in the Manaus programme [23,37,38].

This study has three limitations that must be considered in planning evaluations of nutritional policies: lack of a control group not assisted by a food supplementation programme, which would not be viable or ethical, lack of data on the usual diet of the studied children to observe the possible direct impact on nutritional status and a protocol that provides a reduction in participant loss during follow-up, allowing the inclusion of as much information as possible in the final evaluation of the programme’s effectiveness.

Our study had several strengths. The sample size was sufficient for all statistical analyses, and programme effectiveness was demonstrated using two statistical strategies that provided complementary and concordant results: nutritional indicators of acute malnutrition (wasting), chronic malnutrition (stunting), and overweight. 

## 5. Conclusions

The child nutrition programme promoted by the Municipal Health Office of Manaus is an essential and effective strategy. It aids in the recovery from weight deficits, reduces excess weight, and enhances the H/A indicator in children with short stature.

## Figures and Tables

**Table 1:** Description of maternal and child characteristics, according to sociodemographic data and health conditions of the 7752 children admitted to the programme between 12 and 24 months in Manaus, 2017–2020.

	*n*	%
Maternal characteristics		
Mother’s/guardian’s age (*n* = 7682) ^1^		
<20 years old	594	7.7
≥20 years old	7088	92.3
Number of children (*n* = 5934) ^1^		
≤2	3345	56.4
≥3	2589	43.6
Infant characteristics		
Sex		
Boys	4053	52.3
Girls	3699	47.7
Prematurity history	425	5.5
Supplementing vitamin A	5197	67.0
Supplementing iron	694	9.0
Hospital admission history	169	2.2
Weight and height distribution according to z-scores ^2^		
Weight-for-age z-scores		
Deficit	123	1.6
Adequated	7211	93.0
Excess	418	5.4
Weight-for-height z-scores		
Deficit	104	1.3
Adequated	6616	85.4
Excess	1032	13.3
BMI-for-age z-scores		
Deficit	130	1.7
Adequated	6308	81.4
Excess	1314	16.9
Height-for-age z-scores		
Deficit	980	12.6
Adequated	6772	87.4

^1^ Number of information available in the database. ^2^ Z-scores: deficit (<−2.0 standard deviations); adequated: (−2.0 to +2.0 standard deviations); excess (>+2.0 standard deviations).

**Table 2:** Number of weight and height measurements on admission and follow-ups, according to the age of the 7752 children admitted to the ‘Leite do Meu Filho’ programme between 12 and 24 months in Manaus (2017–2020).

Age (Months)	Admission	Follow-Up of Measurements				Total
		1	2	3	4	
12	168	0	0	0	0	168
13	584	0	0	0	0	584
14	820	0	0	0	0	820
15	878	29	0	0	0	907
16	881	151	0	0	0	1032
17	746	407	4	0	0	1157
18	730	724	50	0	0	1504
19	621	880	134	1	0	1636
20	582	731	227	3	0	1543
21	467	586	277	26	0	1356
22	476	596	347	54	0	1473
23	399	565	376	126	5	1471
24	400	693	565	247	22	1929
25	0	722	574	269	41	1607
26	0	508	429	228	58	1223
27	0	416	404	194	64	1078
28	0	251	307	213	38	813
29	0	179	314	203	47	744
30	0	135	394	192	42	763
31	0	113	288	173	26	600
32	0	37	169	137	29	372
33	0	15	123	103	24	265
34	0	10	57	60	19	146
35	0	1	20	51	14	86
36	0	3	19	37	16	75
Total	7752	7752	5078	2317	445	23,344

**Table 3:** Number and prevalence rates (crude data) of children with a nutritional deficit (<−2 standard-deviations z-score) at admission and during follow-up in the programme.

z-Scores Deficit (z < −2)	*n*	PR	CI 95%	*p* *
W/A Deficit				
Admission (reference)	123	1.00	--	--
1st Follow-up	69	0.56	0.48; 0.66	<0.001
2nd Follow-up	36	0.49	0.39; 0.62	<0.001
3rd Follow-up	14	0.47	0.32; 0.68	<0.001
4th Follow-up	3	0.50	0.22; 1.11	<0.090
W/H Deficit				
Admission (reference)	104	1.00	--	--
1st Follow-up	27	0.26	0.19; 0.31	<0.001
2nd Follow-up	12	0.19	0.11; 0.32	<0.001
3rd Follow-up	7	0.29	0.15; 0.54	<0.001
4th Follow-up	1	0.50	0.12; 2.00	<0.328
BMI/A Deficit				
Admission (reference)	130	1.00	--	--
1st Follow-up	30	0.29	0.21; 0.39	<0.001
2nd Follow-up	11	0.17	0.10; 0.29	<0.001
3rd Follow-up	8	0.33	0.19; 0.58	<0.001
4th Follow-up	1	0.50	0.12; 2.00	0.328
H/A Deficit				
Admission (reference)	980	1.00	--	--
1st Follow-up	465	0.47	0.44; 0.51	<0.001
2nd Follow-up	240	0.36	0.33; 0.40	<0.001
3rd Follow-up	97	0.30	0.26; 0.36	<0.001
4th Follow-up	17	0.26	0.18; 0.40	<0.001

* *p* < 0.05, obtained using Poisson regression with robust variance. PR = Prevalence ratios. 95% CI = 95% of the confidence intervals.

**Table 4:** Mean evolution and standard deviations of z-scores from children admitted with a nutritional deficit (*n* = 7752) using data from admission to the programme as a reference.

Z-Scores	Mean	SD	*p* *
W/A z-scores			
Admission (reference)	−2.50	0.50	--
1st Follow-up	−1.93	1.01	<0.001
2nd Follow-up	−1.97	0.76	<0.001
3rd Follow-up	−1.95	0.67	<0.001
4th Follow-up	−1.47	0.94	0.002
W/H z-scores			
Admission (reference)	−1.72	0.91	--
1st Follow-up	−1.17	1.15	0.000
2nd Follow-up	−1.26	0.92	0.002
3rd Follow-up	−1.38	0.89	0.096
4th Follow-up	−1.09	0.85	0.134
BMI/A z-scores			
Admission (reference)	−1.36	1.11	--
1st Follow-up	−0.85	1.28	0.001
2nd Follow-up	−1.00	1.06	0.035
3rd Follow-up	−1.17	1.00	0.428
4th Follow-up	−0.92	0.91	0.359
H/A z-scores			
Admission (reference)	−2.49	1.18	--
1st Follow-up	−2.13	1.36	0.018
2nd Follow-up	−2.03	1.10	0.010
3rd Follow-up	−1.84	0.97	0.009
4th Follow-up	−1.33	1.43	0.022

* *p* < 0.05, multilevel linear regression; SD = standard deviation.

**Table 5:** Mean z-score differences (W/A, W/H, BMI/A, H/A) at admission and in participants, in the total sample (*n* = 7752), and the nutritional status categories at entry, obtained by multilevel modelling (crude and complete), Manaus, 2017–2020.

	W/A z-Scores		W/H z-Scores		BMI/A z-Scores		H/A z-Scores	
Crude Model	Ratio	95% CI	Ratio	95% CI	Ratio	95% CI	Ratio	95% CI
Total sample								
Constant	0.2556 *	0.2321; 0.2793	0.7034 *	0.6775; 0.7293	0.8298 *	0.8026; 0.8570	−0.5503 *	−0.6182; −0.4824
Admission	Reference		Reference		Reference		Reference	
in the program	−0.0635 *	−0.0764; −0.0506	−0.1831 *	−0.2031; −0.1631	−0.2125 *	−0.2346; −0.1904	0.1496 *	0.1307; 0.1685
With a deficit in admission								
Constant	−2.4942 *	−2.6118; −2.3767	−2.5280 *	−2.6950; −2.3611	−2.5403 *	−2.6983; −2.3821	−2.7128 *	−2.8083; −2.6173
Admission	Reference		Reference		Reference		Reference	
in the programme	0.5738 *	0.4331; 0.7146	1.2889 *	1.0769; 1.5009	1.4073 *	1.1859; 1.6286	0.9371 *	0.8778; 0.9963
Without a deficit in admission								
Constant	0.3038 *	0.2789; 0.3287	0.7528 *	0.7267; 0.7789	0.8877 *	0.8614; 0.9140	−0.2607 *	−0.3066; −0.2148
Admission	Reference		Reference		Reference		Reference	
in the programme	−0.0739 *	−0.0867; −0.0611	−0.2025 *	−0.2214; −0.1836	−0.2442 *	−0.2660; −0.2222	0.0428	0.0242; −0.0615
Complete Model	Ratio	95% CI	Ratio	95% CI	Ratio	95% CI	Ratio	95% CI
Constant	0.4301 *	0.3932; 0.4670	0.8658 *	0.8205; 0.9165	0.9674 *	0.9160; 1.0188	−0.1533 *	−0.2141; −0.0925
Admitted with deficit								
No (reference)	Reference		Reference		Reference		Reference	
Yes	−3.1419 *	−3.4125; −2.8713	−3.5422 *	−3.9241; −3.1603	−2.5838 *	−2.9067; −2.2608	−2.7015 *	−2.8245; −2.5785
Status in the programme								
Admission (reference)	Reference		Reference		Reference		Reference	
In the programme	0.0694 *	0.0196; 0.1193	0.2211 *	0.1447; 0.2975	0.2558 *	0.1716; 0.3399	−0.2244 *	−0.2693; −0.1796
Program × Deficit	0.7073 *	0.4604; 0.9543	1.2549 *	0.8570; 1.6528	1.2106 *	0.7754; 1.6458	0.5910 *	0.4648; 0.7172
Age in the follow-up	−0.0246 *	−0.0314; −0.0178	−0.0269 *	−0.0366; −0.0172	−0.0205 *	−0.0311; −0.0100	0.0177 *	−0.0238; −0.0117
Age × Deficit	0.0570 *	0.0231; 0.0910	0.0464	−0.0039; 0.0967	−0.0141	−0.0614; 0.0332	0.0452 *	0.0283; 0.0621
Age squared	0.0004 *	0.0001; 0.0008	0.0008 *	0.0002; 0.0013	0.0006 *	0.000; 0.0012	--	--
Program × Age	−0.0046	−0.0019; 0.0006	−0.0315 *	−0.0403; −0.0227	−0.0397 *	−0.0494; −0.0300	0.0323 *	0.0267; 0.0378
Program × Age × Deficit	−0.0317 *	−0.0061; −0.0025	0.0024	−0.0480; 0.0431	0.0354	−0.0114; 0.0822	0.0022	−0.0132; 0.0177

* *p* < 0.05, obtained using multilevel linear regression. Coef = Regression coefficient. 95% CI = 95% confidence intervals. Program status: admission (entry into the programme) = 0; in the programme (during the intervention) = 1. Program × Deficit = interaction variables between variable programme (0 or 1) and variable nutritional deficit on admission (NDA: 0 or 1). Age at follow-up = age at measurement (in months). Age × Deficit = multilevel regression z-score units for each month of age increase in infants admitted with deficits. Age squared = age at follow-up (in months) squared. Programme × Age = interaction variable between programme variables (0 and 1) and age at follow-up. Programme × Age × Deficit = interaction variable between programme variable (0 and 1), age at follow-up, and variable ‘nutritional deficit upon entry’ (DNE: 0 or 1).

**Table 6:** Z-scores paired evolution at admission and after 6–9 months of intervention in the evaluated group and according to the nutritional deficit at admission, Manaus, 2017–2020.

Z-Scores	*n*	Admission	After 6–9 Months of Follow-Up	Difference between Admission and 6–9 Months Follow-Up	*p* *
Admitted without deficit					
Weight for age	1599	+0.38 (0.96)	+0.27 (0.97)	−0.11	<0.001
Weight for height	1601	+0.80 (1.14)	+0.66 (1.17)	−0.14	<0.001
BMI for age	1592	+0.92 (1.20)	+0.78 (1.23)	−0.14	<0.001
Height for age	1412	−0.19 (1.10)	−0.31 (1.11)	−0.12	<0.001
Admitted with deficit					
Weight for age	18	−2.47 (0.43)	−1.79 (0.90)	+0.68	0.013
Weight for height	16	−2.60 (0.49)	−1.13 (1.74)	+1.47	0.004
BMI for age	25	−2.53 (0.57)	−0.92 (1.89)	+1.61	<0.001
Height for age	205	−2.60 (0.64)	−1.64 (1.17)	+0.96	<0.001

* Mean (standard deviation), paired *t*-test; BMI: body mass index.

**Table 7:** Mean value comparison of body mass index-for-age z-scores on admission and during programme permanence (after 6–9 months), according to nutritional status upon entering the programme, Manaus, 2017–2020.

	*n* (%)	Admission	Intervention for 6–9 Months	Mean (SD)	*p* *
Wasting (z < −2.0 SD)	25 (1.6)	−2.53 (0.57)	−0.92 (1.89)	+1.61	<0.001
Eutrophic (z between −2.0 and +1.0 SD)	868 (53.7)	+0.04 (0.70)	+0.26 (1.10)	+0.22	<0.001
Overweight risk (z between +1 and +2 SD)	417 (25.8)	+1.45 (0.27)	+1.18 (1.00)	−0.27	<0.001
Overweight (z between +2.0 and +3.0 SD)	232 (14.3)	+2.41 (0.28)	+1.63 (1.06)	−0.78	<0.001
Obesity (z > +3.0 SD)	75 (4.6)	+3.56 (0.50)	+2.33 (1.18)	−1.23	<0.001

* Mean (standard deviation), paired *t*-test; BMI: body mass index.

## Data Availability

Data is contained within the article.

## Supplementary Materials

The following supporting information can be downloaded at: https://www.mdpi.com/article/10.3390/nu16071051/s1, Figure S1: Sample selection process flowchart; Table S1: Nutritional composition of the infant formula used as a food supplement for children aged 12–24 months in the program “Leite do Meu Filho” (Manaus, 2017–2020).

## References

[1] FAO, UNICEF, WFP, WHO (2021). The State of Food Security and Nutrition in the World: Transforming Food Systems for Food Security, Improved Nutrition, and Affordable Healthy Diets for All.

[2] Nogueira-de-Almeida C.A., Ribas Filho D., Weffort V.R.S., Ued F.V., Nogueira-de-Almeida C.C.J., Nogueira F.B., Steiner M.L., Fisberg M. (2022). Primeiros 2.200 dias de vida como janela de oportunidade de atuação multidisciplinar relativa à origem desenvolvimentista de saúde e doença: Posicionamento da Associação Brasileira de Nutrologia. IJN.

[3] World Health Organization (WHO) Breastfeeding. https://www.who.int/health-topics/breastfeeding#tab=tab_2.

[4] Duale A., Singh P., Al Khodor S. (2021). Breast milk: A meal worth having. Front. Nutr..

[5] Sociedade Brasileira de Pediatria (2021). Temas da Atualidade em Nutrologia.

[6] Universidade Federal do Rio de Janeiro (2020). Estudo Nacional de Alimentação e Nutrição Infantil: ENANI-2019: Resultados Preliminares: Indicadores de Aleitamento Materno no Brasil.

[7] WHO, UNICEF (2003). Global Strategy for Infant and Young Child Feeding.

[8] WHO (2006). Multicentre Growth Reference Study Group. Breastfeeding in the WHO multicentre growth reference study. Acta Paediatr. Suppl..

[9] IBGE Censo Demográfico 2020. https://www.ibge.gov.br/censo2020.

[10] Alencar F.H., Yuyama L.K.O., Rodrigues E.F.R., Esteves A.V.F., Mendonça M.M.B., Silva W.A. (2008). Magnitude da desnutrição infantil no Estado do Amazonas/AM—Brasil. Acta Amaz..

[11] Carvalho C.A., Fonseca P.C.A., Priore S.E., Franceschini S.C.C., Novaes J.F. (2015). Consumo alimentar e adequação nutricional em crianças brasileiras: Revisão sistemática. Rev. Paul. Pediatr..

[12] Araujo T.S., Oliveira C.S.M., Muniz P.T., Silva-Nunes M., Cardoso M.A. (2016). Desnutrição infantile m um dos municípios de maior risco nutricional do Brasil: Estudo de base populacional na Amazônia Ocidental Brasileira. Rev. Bras. Epidemiol..

[13] Tavares B.M., Veiga G.V., Yuyama L.K.O., Bueno M.B., Fisberg R.M., Fisberg M. (2012). Estado nutricional e unicip de energia e unicipio de pré escolares que frequentam creches no unicipio de Manaus, Amazonas: Existem diferenças entre creches públicas e privadas?. Rev. Paul. Pediatr..

[14] Dulal S., Prost A., Karki S., Saville N., Merom D. (2021). Characteristics and effects of integrated nutrition and stimulation interventions to improve the nutritional status and development of children under 5 years of age: A systematic review and meta-analysis. BMJ Glob. Health.

[15] Matsuyama M., Harb T., David M., Davies P.S., Hill R.J. (2017). Effect of fortified milk on growth and nutritional status in young children: A systematic review and meta-analysis. Public Health Nutr..

[16] Eichler K., Wieser S., Rüthemann I., Brügger U. (2012). Effects of micronutrient fortified milk and cereal food for infants and children: A systematic review. BMC Public Health.

[17] Mannar M.G. (2006). Successful food-based programs, supplementation, and fortification. J. Pediatr. Gastroenterol. Nutr..

[18] Vessoni A.T., Jaime P.C. (2019). Programas de suplementação alimentar com leite e a agenda de segurança alimentar e nutricional brasileira. Demetra.

[19] Castro I.R.R., Monteiro C.A. (2002). Avaliação do impacto do programa “Leite é Saúde” na recuperação de crianças desnutridas no Município do Rio de Janeiro. Rev. Bras. Epidemiol..

[20] Magalhães R. (2014). Evaluation of public policies and initiatives in food and nutrition security: Dilemmas and methodological perspectives. Ciên Saúde Colet..

[21] Pacheco Santos L.M., Araújo M.D., Martins M.C., Veloso I.S., Assunção M.P., Chaves dos Santos S.M. (2007). Evaluation of Brazilian public policies to promote food security and fight hunger, 1995–2002. 2—The Workers’ Nutrition Program. Cad. Saude Publica.

[22] Valle N.J., Santos I.S., Gigante D.P. (2004). Nutritional interventions and child growth among under-two-year-olds: A systematic review. Cad. Saude Publica.

[23] Augusto R.A., Souza J.M.P. (2010). Effectiveness of a supplementary feeding program in child weight gain. Rev. Saude Publica.

[24] Tonete V.L.P., Carvalhaes M.A.B.L., Trezza E.M.C. (2013). Evolução nutricional de crianças carentes atendidas por programa de suplementação alimentar. Pediatria.

[25] WHO (2006). WHO Child Growth Standards-Methods and Development: Length/Height-for-Age, Weight-for-Age, Weight-for-Length, Weight-for-Height and Body Mass Index-for-Age.

[26] Organização das Nações Unidas para a Agricultura e Alimentação (FAO) (1996). Declaração de Roma Sobre a Segurança Alimentar Mundial & Plano de Ação da Cúpula Mundial da Alimentação. Cúpula mundial de alimentação.

[27] Goulart R.M., França Junior I., Souza M.d.F. (2007). Nutritional rehabilitation of undernourished and nutritionally at-risk children admitted to a supplementary food program in Mogi das Cruzes, São Paulo, Brazil. Cad. Saude Publica.

[28] Goulart R.M.M., França Júnior I., Souza M.F.M. (2009). Fatores associados à recuperação nutricional de crianças em programa de suplementação alimentar. Rev. Bras. Epidemiol..

[29] Cardoso J.L., Pires M.M.S., Pinheiro C.E., Nassar S.M. (2010). Recuperação nutricional em programa de suplementação alimentar infantil em Florianópolis. Acad. Med..

[30] Pranzl M.A., Oliveira N.R.F. (2013). O uso de fórmulas lácteas e o perfil nutricional de crianças atendidas por um programa municipal de combate às carências. Disciplinarum Sci..

[31] Santos I.S., Gigante D.P., Coitinho D.C., Haisma H., Valle N.C.J., Valente G. (2005). Evaluation of the impact of a nutritional program for undernourished children in Brazil. Cad. Saude Publica.

[32] Gutierrez M.R., Bettiol H., Barbieri M.A. (1998). Evaluation of a supplementary nutrition program. Rev. Panam. Salud Publica.

[33] Carvalho L.G., Saldiva S.R.D.M., Rosa T.E.C., Lei D.L.M. (2009). Evolução do estado nutricional de crianças submetidas a um programa de suplementação alimentar em município do Estado de São Paulo. Rev. Nutr..

[34] Organización de las Naciones Unidas Para la Agricultura y la Alimentación (FAO) (1993). Directrices Para la Formulación de Planes Nacionales de Acción para la Nutrición. Conferência Internacional Sobre Nutrição.

[35] Duarte M.G., dos Santos S.F., Minatto G., Nobre G.C., dos Santos J.O., de Sousa T.F., Junior I.F. (2018). Estado nutricional de crianças do baixo Amazonas: Concordância entre três critérios de classificação. J. Hum. Growth Dev..

[36] Nogueira-de-Almeida C.A., Falcão M.C., Ribas-Filho D., Zorzo R.A., Konstantyner T., Ricci R., Gioia N., Fisberg M. (2020). Consensus of the Brazilian association of Nutrology on milky feeding of children aged 1–5 years old. Int. J. Nutrol..

[37] Sguassero Y., De Onis M., Bonotti A.M., Carroli G. (2012). Community-based supplementary feeding for promoting the growth of children under five years of age in low- and middle-income countries [Review]. Cochrane Database Syst. Rev..

[38] Ministério da Saúde: Brasil. Secretaria de Atenção à Saúde (2014). Aleitamento Materno, Distribuição de Leites e Fórmulas Infantis em Estabelecimentos de Saúde e a Legislação.

