# Supplementary material for: Improving the Nutritional Status of Socially Vulnerable Children in Manaus, Brazilian Amazon, through a Food Supplementation Programme
Source: Nutrients. 2024 Apr 4;16(7):1051. doi: 10.3390/nu16071051 (PMC11013924; doi:10.3390/nu16071051)
Supplement: Supplementary file 1 [file nutrients-16-01051-s001.zip › nutrients-2819674-supplementary.pdf]

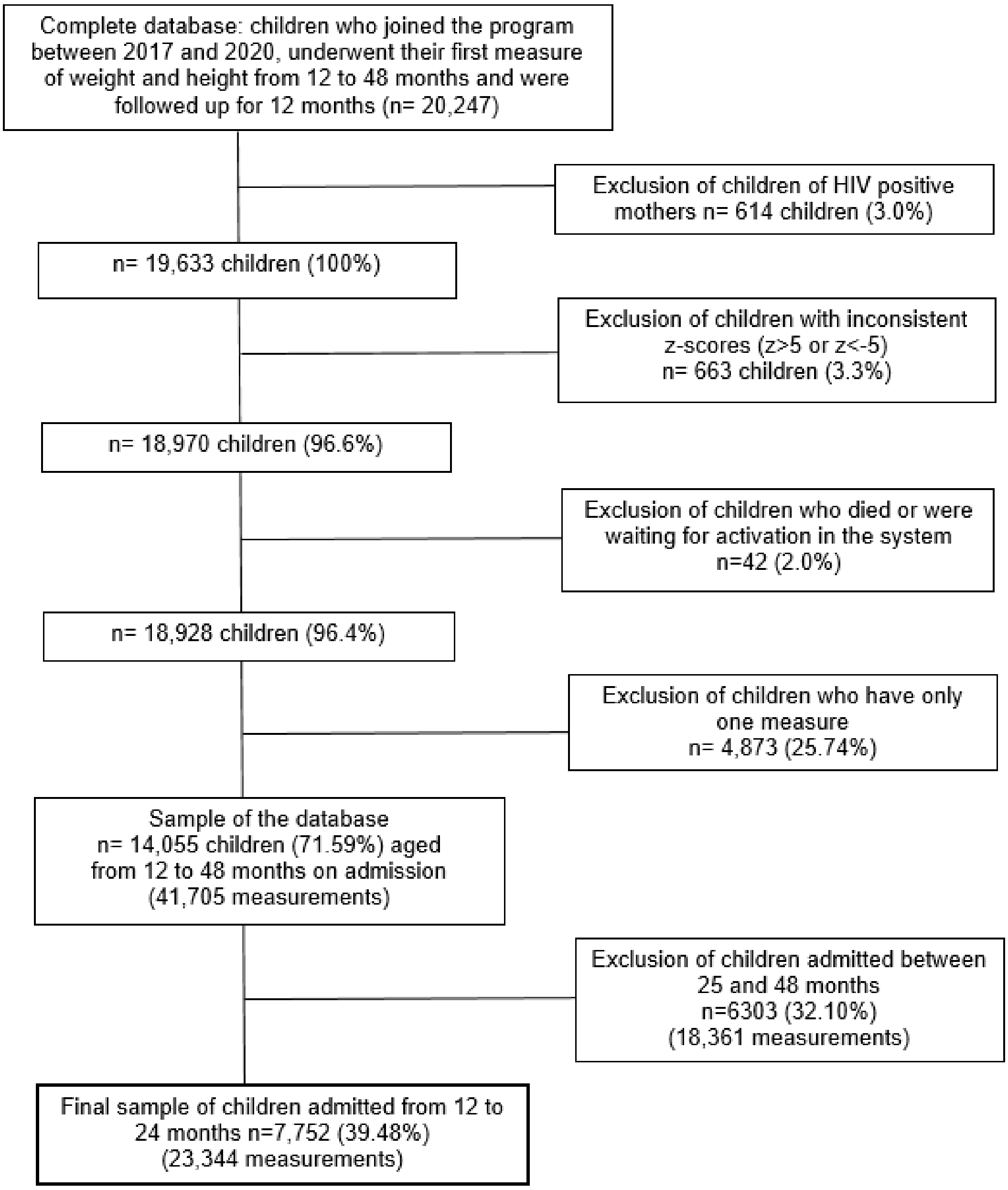

Figure S1. Sample selection process flowchart.

Table S1. Nutritional composition of the infant formula used as a food supplement for children aged 12–24 months in the program "Leite do Meu Filho" (Manaus, 2017–2020).

Energy and Nutrient Value	Unit	In 100g of powder	In 100 ml of reconstituted formula
Energetic value	kcal	472	67
Energetic value	kJ	1982	281
Carbohydrates	g	58	8.3
Lactose	g	44	6.2
Proteins	g	11	1.5
Total fat	g	22	3.1
Saturated fat	g	8.0	1.1
Trans fats	g	0	0
Linoleic acid	g	3.1	0.4
α-linolenic acid	mg	380	54
Dietary fiber, of which:	g	2.9	0.4
Galactooligosaccharides (GOS)	g	2.6	0.4
Fructooligosaccharides (FOS)	g	0.29	0.04
Sodium	mg	240	34
Calcium	mg	550	78
Iron	mg	7.8	1.1
Potassium	mg	625	89
Chloride	mg	320	45
Phosphor	mg	310	44
Magnesium	mg	60	8.5
Iodine	µg	150	21
Copper	µg	360	51
Zinc	mg	5.6	0.80
Selenium	µg	11	1.6
Manganese	µg	60	8.5
Vitamin A	Mg RE	570	81
Vitamin D	µg	11	1.5
Vitamin E	mg α TE	11	1.5
Vitamin K	µg	45	6.4
Vitamin C	mg	75	11
Vitamin B1	mg	1.0	0.14
Vitamin B2	mg	1.2	0.17
Niacin	mg	5.0	0.71
Vitamin B6	mg	0.58	0.08
Folic acid	µg	130	18
Pantothenic acid	mg	6.4	0.91
Vitamin B12	µg	1.5	0.21
Biotin	µg	24	3.4
Choline	mg	80	11
Inositol	mg	35	5.0
